# Prof. Beale and His Doctrines

**Published:** 1870-12

**Authors:** I. N. Danforth

**Affiliations:** Chicago


### Article VI.—*Prof. Beale and his Doctrines.* By I. N. Danforth, M.D., Chicago.

No account of the rise and progress of cell doctrines, however meagre, can be considered complete which does not embrace a review of the labors and views of Prof. Lionel S. Beale, of London. It is, I think, perfectly safe to affirm, that Prof. Beale is the most accomplished microscopist living; and as this is an era of skillful microscopists, it is probably equally safe to say, that no other microscopist has ever equaled him—not even excepting Quekett and Goodsir. He is peculiarly expert in the delicate manipulations necessary to the satisfactory, or at least profitable, use of the microscope; and well he may be, for his skill is the outgrowth of long years of such devoted labor as can only be expected of an ardent enthusiast, who is fully impressed with the belief that he has "work to do in the world."

Prof. Beale has also greatly extended our knowledge as to modes of preparing tissues for examination with high powers, staining tissues, making fine injections, and successfully preserving the most delicate textures for long periods of time. He has made a very patient, laborious, careful, and, I may add, highly successful, study of the use of the highest powers, and their adaptability to investigations upon all classes of healthy and morbid tissues. Indeed, he may be regarded as the true originator of high powers, as the term is understood at the present day.

Up to the year 1860, the sixteenth-inch objective was the highest power in use, and its employment was necessarily restricted to a very few of the more expert microscopists. In 1859, Dr. Beale desired Messrs. Powell & Lealand to attempt the construction of a glass "with a magnifying power double that of the sixteenth," and this they succeeded in producing the following year; it being the "first twenty-sixth ever made." Its magnifying power is 1800 diameters, and its value as a "working glass" was found by Prof. Beale to be very great. Encouraged by his success, he again applied to Powell & Lealand to manufacture an objective of still greater power; and, in 1864, in the month of October, they completed a fiftieth-of-an-inch objective, with an amplifying power of 3,000 diameters. Those who are practically conversant with the use of the microscope, will at once understand the difficulties which had to be overcome in the production of this "wonderful glass," as Dr. Beale so justly calls it. They will also call to mind the exquisite delicacy of manipulation requisite for the successful preparation of specimens to be examined therewith. Concerning its qualities as a "working glass," Dr. Beale says, "it defines quite as well as the twenty-fifth, and there is no difficulty in obtaining plenty of light for the illumination of the objects." (How to Work with the Microscope, page 286.)

Until the ice was thus broken by Beale, it was supposed to be practically impossible to use objectives of such immense power, on account of the presumed impossibility of focussing, and of obtaining illumination and defining power; and it is a somewhat unaccountable and very mortifying fact, that the pioneer in this most praiseworthy movement, was immediately assailed and ridiculed by several of his cotemporaries, in London and elsewhere, for attempting to accomplish that which could *not* be accomplished. Such extremely high powers, however, are never likely to come into very general use, both on account of their very great expense, and the extreme difficulty of working with them.

But Dr. Beale has conferred an infinitely greater service on the world of microscopists, by the introduction of a very simple and equally efficient manner of preparing tissues for examination. This method consists, essentially, in saturating the specimens with the purest and strongest glycerine, to which has been added a small proportion of acetic acid; and in describing the details of the process, so clearly and intelligibly, that the merest tyro can prepare and preserve his own specimens. After a somewhat extended experience in the preparation and study of the tissues, by Dr. Beale's method, I am quite convinced that it is much superior to any other with which I am acquainted.

The carefully-written and elegantly-illustrated works of Prof. Beale are not so generally sought as they should be; two of them, however, namely, "How to Work with the Microscope," and "The Microscope in Practical Medicine," have met with quite an extensive sale in this country and abroad. But his "Archives," "Kidney Diseases," and other works, are not so generally found in the libraries of physicians. All of these works are beautifully and profusely illustrated. In the hands of their author, the art of making drawings of microscopic appearances has assumed a new phase entirely.

Having thus cursorily glanced at what Dr. Beale has accomplished for microscopy as a branch of medical science, we are now prepared to examine his peculiar views regarding the cell and its functions.

In the first place, it is but just to state, that Dr. Beale's theories are not the expression of a mere *hobby;* nor have they been constructed with the intention of bolstering up any preconceived notions regarding the nature of vitality or its origin. When he first commenced his remarkable microscopical studies and observations, he had no theories peculiarly his own. His present views, therefore, are but the legitimate and necessary outgrowth of his laborious and persistent observations upon the tissues, at all stages, both of their development and their disintegration.

At the very outstart, we find that Beale practically repudiates the old notion of the "type cell," which was looked upon as "an extremely monotonous structure," (Virchow), consisting essentially of *cell wall*, *contained fluid*, *granules*, *and nucleus*. According to the old theory, the cell wall was considered not only a very *important* part of the cell, but the part which maintained the nearest and truest relations with vitality. According to Beale, the cell wall is not only unimportant, but very often absent; and, when present, is merely the result of secondary changes, occurring upon the surface of the cell. "All that is essential to the cell, or elementary part, is *matter that is in the living state—germinal matter*, and matter that *has been in the living state—formed material.*" (Protoplasm, page 55.)

In studying Dr. Beale's views, it will be most convenient to take up successively the so-called "nucleus," or germinal matter, and the surrounding cell-contents, or "formed material."

*First*—the nucleus. Prof. Beale believes that the nucleus is the only portion of the cell which is in the "living state;" it is the true ultimate repository of vital power, and therefore, practically, the ultimate anatomical living element. This body is always and everywhere easily distinguishable from the older, non-living portions of the cell—the so-called "formed material"—by its property of being readily stained by an ammoniacal solution of carmine. This discovery was announced by Prof. Beale several years since, and it has since been abundantly confirmed by a multitude of observers in different parts of the world. Indeed, it is now quite generally recognized by microscopists as a reliable test for the purpose of distinguishing the nucleus, or "germinal matter," from the proper cell-contents, or "formed material." The relative size of the nucleus depends upon the age of the cell, and the accessibility of nutrient material. In young cells, situated quite near their source of pabulum, as the lower stratum of epithelial or epidermic cells, the amount of germinal or living matter is very large, forming, at the *commencement* of their development, their *only* constituent. As they approach the surface, and recede from their source of nutrition, the "germinal matter" decreases, while the "formed material" increases. The same rule holds good in all varieties of cell growth, whether healthy or morbid—whether animal or vegetable. In his earlier lectures on the structure and growth of the tissues, (Archives of Medicine, vol. II,) Prof. Beale states, that this germinal matter is "granular" in its structure; in his recent publications, he states that it is "*clear*, *transparent*, *structureless*, *formless*," (Protoplasm, p. 48.) This apparent change of opinion is to be accounted for by the fact that Dr. Beale has recently very greatly increased his powers of observation, by the acquisition of the fiftieth-of-an-inch objective already alluded to.

According to the views which I have attempted to explain, the nucleus or germinal matter is the essential part of the cell; it is the mysterious seat of vitality — the repository of life, the ultimate, indivisible, anatomical element. All vital actions, of whatever nature, depend upon the forces lodged within this simple and very minute body. Assimilation, secretion, locomotion, reproduction and thought depend, directly or indirectly, upon the exercise of the powers locked up in this infinitesimal speck of living matter. Its power of growth is practically without limit. So long as the germinal matter is supplied with a sufficient amount of appropriate pabulum, it grows and subdivides indefinitely. Each subdivision of germinal matter—each little mass which is thrown off from the parent mass, is endowed with all the powers and capabilities of the parent mass; thus each centre of growth, or nucleus, *may*—and if favorably situated and adequately nourished, *does* become, the source of very many new centres of growth. Very recently, Prof. Beale has conferred upon the germinal matter the name of "*Bioplasm*" as a substitute for the word "*Protoplasm*," which is already loaded down with more theories than it can conveniently carry. Regarding the use of this word, Dr. Beale says: "The term I propose to apply to the *living* or *germinal self-propagating* matter of living beings, and to restrict to this, is *Bioplasm.* Now that the word *Biology* has come into common use, it seems desirable to employ the same root in speaking of the matter which it is the main purpose of biology to investigate. *Bioplasm* involves no theory as regards the nature or the origin of the matter. It simply distinguishes it as *living.* A living white-blood corpuscle is a mass of *bioplasm*, or it might be termed a *bioplast.* A very minute living particle is a bioplast, and we may speak of living matter as bioplasmic substance." ("Disease Germs," page 12.)

*Secondly:* the "formed material." Formed material constitutes all that part of the cell which *is not* stained by the action of the carmine solution; and it is a remarkable fact, that, although the carmine fluid must necessarily *pass through* the formed material, in order to come in contact with the germinal matter, the former does not become tinted thereby. But formed material is not by any means limited to the proper "cell contents," (periplastic substance of Huxley.) The so-called intercellular substance, which finds its most perfect type in cartilage, is also formed material; while the cartilage cells are chiefly composed of bioplasm, or living matter, from whence is derived the newly-formed "material" for the repair of cartilage. The same rule holds good as regards every other tissue; every muscle, every bone, every ligament, every gland, consists largely of "formed material," but at the same time we find myriads of minute masses of germinal matter, which are constantly assimilating new pabulum, constantly growing, constantly dividing, and constantly being converted into "formed material," to supply the place of that which is being worn out and converted into effete matter. Thus every atom of nutrient matter is converted first into germinal matter, (living matter, bioplasm) and next into formed material. Following this doctrine out to its practical application, we find that mucus is the *formed material* of the mucous corpuscle, the bile the *formed material* of the hepatic cell, the muscular fibril the *formed material* of the muscle cell, and the ultimate nerve fibre the *formed material* of the nerve cell. In other words, it is the proper function of "germinal matter" to convert pabulum first into other "germinal matter" like itself, and then into tissue like that of which it forms an integral part. That which was yesterday lifeless pabulum, is to-day living "germinal matter;" that which to-day is germinal matter, will to-morrow be formed material. As the formed material is increased at the expense of the germinal matter, the latter must of course decrease in exact proportion to the increase of the former; in other words, just *as* fast as *living* germinal matter decreases, just *so* fast does *non-living* formed material increase. Thus the formed material is made to subserve the purpose of a protective coating or envelope to the living matter or germ, shielding it from the destructive influences of heat, cold and moisture, until it shall, perchance, reach its appropriate *nidus* for future growth and development. This fact is peculiarly illustrated in the ovum of the tape worm, which after various vicissitudes and exposures, at last finds itself favorably situated for manifesting the developmental powers which have, for a longer or shorter time, been in a state of latent vitality.

Prof. Beale holds that the formed material is non-living matter; that it has, to all intents and purposes, ceased to live; but his test of vitality is the presence or absence of the *power of germination or reproduction.* This idea is very clearly expressed in Beale's edition of Todd and Bowman's Physiology, Part I, page 12, namely, "the *entire cell* or elementary part is not alive. The inner (germinal) matter is living—the outer formed matter has ceased to live. The inner matter alone is capable of growth, of germination. It may, therefore, be called living or germinal matter, in contradistinction to the passive formed matter which envelops it." Again, he says, "the difference between germinal or living matter and the pabulum which nourishes it, on the one hand, and the formed material which is produced by it on the other, is, I believe, absolute. * * * * The ultimate particles of matter pass from the lifeless into the living state and from the latter into the dead state suddenly. Matter cannot be said to *half live* or *half die.* It is either *dead* or *living*, *animate* or *inanimate;* and formed matter has ceased to live." ("Protoplasm, or Life, Matter and Mind," page 36.) I think the great majority of histologists will find themselves compelled to dissent from this conclusion, if the term *dead* is to be used in its ordinary acceptation. It is simply equivalent to saying that a large proportion of every tissue and organ of the human body has ceased to live. For my own part, while I cannot claim that the mucus, or the bile, or the hair, or the free extremities of the nails, or the outer layer of the epidermis, consist of living matter, I certainly cannot admit that the muscular substance of my heart, or the nerve fibrils which convey external impressions to my sensorium, or the delicate membrane which lines my blood vessels, and in great part forms the capillaries of my body, are, either or any of them, composed essentially of dead matter. I do not think Dr. Beale means, or would claim this. It is much more probable that he intends to use the term "dead" in an arbitrary, technical sense, and as a convenient way of expressing the difference in developmental power between germinal matter and formed material. Nor do I think it quite just to conclude that, because one portion of a muscle can no longer assimilate nutrient matter and produce other material like itself, it has therefore ceased to live. Would it not be just *as* correct to say that an octogenarian, in whom the procreative power had passed away, had therefore ceased to live? If the presence or absence of the power of reproduction is really the test of the presence or absence of life, is not the conclusion as fair and as logical in the one case as in the other? The term "dead," "nonliving" or "lifeless" matter, as applied to formed material or the non-germinal portion of the solid tissues, is, it seems to me, a most unfortunate one. It can scarcely do otherwise than mislead very many of those, at least, who are commencing their medical studies, while it will be very apt to confuse and pester those who are more acccustomed to groping their way among the pitfalls of medicine. Let any practiced observer prepare a specimen of frog's muscle, and properly stain it with carmine, and then carefully examine it with a fifth or eighth-inch objective; he will find himself forced to the conclusion that if *only* the minute masses of "germinal matter" are *living*, the frog must have a wonderful capacity for jumping by the aid of dead muscles. We need not value Prof. Beale's microscopic observations any the less, however, merely because some of the conclusions which he draws from them are not quite acceptable.

Prof. Beale is a vigorous and outspoken opponent of the doctrine—at the present time so loudly trumpeted—of that small, but rather select school of "advanced thinkers" in England, who are assiduously laboring to place vital force and chemical force on the same level. Within the past few years, this species of heresy has taken a new lease of life, and clothed itself in new apparel. "Protoplasm," in assuming to be the "physical basis of life," has attempted to eject vitality, *per se*, from the organic world; "spontaneous generation" is re-enacting the old farce, under the auspices of new and not less skillful performers; but, from whatever stand-point vital force has been assailed, it has found in Prof. Beale a steady, unflinching and able advocate. On this subject, the writings of Dr. Beale are models of controversial labor. In conclusion, the essence of the doctrines of Beale may be briefly summarized in the following manner:

*First:* the *germinal matter*, nucleus, bioplasm, (endoplast of Huxley) is the essential, potential, vital and active portion of the cell; it is the ultimate repository of vitality, and wherever a mass of germinal matter exists, however minute it may be, or whatever form it may assume, *there* a cell exists, for the germinal matter is, practically, the cell.

*Secondly:* the *formed material*, cell contents, (periplast of Huxley) is matter which has resulted from changes incident to the death of the germinal matter; when the latter dies it becomes formed material; all formed material *was* germinal matter; all germinal matter *will be* formed material. Formed material is chiefly concerned in the *function* of the tissue or organ whereof it forms a part; while germinal matter is chiefly if not exclusively concerned in reproduction and repair. And the distinction between these two forms of matter seems to be absolute; the dividing line is always sharply drawn; there is no neutral ground between them; or, as Dr. Beale expresses it, "matter cannot be said to *half live* or *half die.*"

*Thirdly:* the chemical and physical views of life (to which I have already alluded) are utterly insufficient to account for the phenomena of life, or indeed any one of them; "spontaneous generation" is likewise equally baseless and without substantial proof. On this latter point, the following passage is sufficiently explicit: "it must then be regarded as a fact that living beings spring from pre-existing living beings, and that there is no such thing as spontaneous generation." ("Protoplasm," page 75.) In this connection, it is proper and very gratifying to remark that Prof. Huxley, in his recent address before the "British Association,' takes the strongest possible ground against the doctrine of "spontaneous generation."

The doctrines of Beale, considered as a whole, have found favor with many, if not most, of our most thoughtful and laborious histologists; and whatever of error may or may *not* be contained in his theories concerning cell-action, he has certainly greatly advanced our knowledge of structure. Moreover, I think he is clearly entitled to the credit of having taught us, more than any other man, how to properly and profitably "work with the microscope," as an aid to diagnosis, and the practical study of pathology; and for this, if for nothing else, he deserves the thanks and gratitude, not of microscopists alone, but of the whole profession.

I have attempted in this article, and those which have preceded it, to give a somewhat complete, though necessarily very brief, history of the rise and progress of the more prominent cell doctrines up to the present time. In doing this, I have already disclaimed, and hereby distinctly disclaim any title to originality, either of thought or method; and I have tried to be somewhat careful in indicating the sources of my information. My only object has been to place before the readers of the "Journal" information for which I had myself been seeking, and very much felt the need of, in the simplest and most concise manner. In future articles I purpose taking up some other topics, holding a near relation to cytology.

